# HD-ZIP IV Gene *ROC1* Regulates Leaf Rolling and Drought Response Through Formation of Heterodimers with ROC5 and ROC8 in Rice

**DOI:** 10.1186/s12284-024-00717-9

**Published:** 2024-07-27

**Authors:** Zhihuan Tao, Xuexia Miao, Zhenying Shi

**Affiliations:** 1 Key Laboratory of Plant Design, CAS Center for Excellence in Molecular Plant Sciences, Institute of Plant Physiology and Ecology, Chinese Academy of Sciences, Shanghai, 200032 China; 2 University of Chinese Academy of Sciences, Beijing, 100049 China

**Keywords:** HD-Zip IV, *ROC1*, Leaf rolling, Bulliform cells, Drought stress response

## Abstract

Leaf morphology is a crucial agronomic characteristic of rice that influences crop yield directly. One primary cause of rice leaf rolling can be attributed to alterations in bulliform cells. Several *HD-ZIP IV* genes have been identified to be epidemical characterized and function in leaf rolling in rice. Still others need to be studied to fully understand the overall function of HD-ZIP IV family. Among the nine *ROC* genes encoding HD-ZIP IV family transcription factors in rice, *ROC1* exhibits the highest expression in the leaves. Overexpression of *ROC1* decreased the size of bulliform cells, and thus resulted in adaxially rolled leaves. To the contrary, knockout of *ROC1* (ROC1KO) through Crispr-cas9 system enlarged bulliform cells, and thus led to abaxially rolled leaves. Moreover, ROC1KO plants were sensitive to drought. ROC1 could form homodimers on its own, and heterodimers with ROC5 and ROC8 respectively. Compared to ROC1KO plants, leaves of the *ROC1* and *ROC8* double knocked out plants (ROC1/8DKO) were more severely rolled abaxially due to enlarged bulliform cells, and ROC1/8DKO plants were more drought sensitive. However, overexpression of *ROC8* could not restore the abaxial leaf phenotype of ROC1KO plants. Therefore, we proved that ROC1, a member of the HD-ZIP IV family, regulated leaf rolling and drought stress response through tight association with ROC5 and ROC8.

**Supplementary Information:**

The online version contains supplementary material available at 10.1186/s12284-024-00717-9.

## Introduction

Rice holds significant importance as a staple food crop globally, with over 50% of the global population depending on it (Sasaki and Burr 2000). Leaf morphology is a significant agricultural characteristic of rice, with its effect extending to leaf photosynthesis, respiration, and transpiration. A moderate rolling of the rice leaves increases the light-receiving space and light transmission rate and light saturation point without affecting the light compensation point, and results in a well-proportioned leaf area for photosynthesis and thus augments yield (Zhang et al. 2009). The mechanism for leaf rolling in gramineae pertains primarily to alterations in the bulliform cells, which are highly specialized epidermal parenchyma cells on the adaxial side of leaf surface in graminaceous plants (Sylvester et al. 2001)**.** Genes regulating bulliform cells development in rice would ultimately influence leaf rolling. For example, *BRD1* (Hong et al. 2002), *AGO7* (Shi et al. 2007), *ADL1* (Hibara et al. 2009), *LC2* (Zhao et al. 2010), *ROC5* (Zou et al. 2011), *SRL1* (Xiang et al. 2012), *LBD3-7* (Li et al. 2016a), *Hox32*/*HB4* (Li et al. 2016b), *RRK1* (Ma et al. 2017) and *ROC8* (Sun et al. 2020) exert a negative influence on the developmental process of bulliform cells, while *NAL7* (Fujino et al. 2008), *ACL1* (Li et al. 2010), *ACL2* (Li et al. 2010), *NRL1*/*CslD4* (Hu et al. 2010), *RL14* (Fang et al. 2012), *ZHD1* (Xu et al. 2014), *ZHD2* (Xu et al. 2014) and *miR166* (Zhang et al. 2018) act positively to promote the development of bulliform cells. Apart from bulliform cells, various other factors contribute to the rolling of rice leaves. *SLL1* plays a role in leaf morphology by regulating programmed cell death of abaxial mesophyll cells (Zhang et al. 2009). What’s more, *CFL1* regulates leaf rolling through influencing cuticle development (Wu et al. 2011).

Drought stress poses a significant limitation to agricultural production on a global scale, primarily as a result of the intricate nature of water scarcity and the impacts of climate change on the environment (Fang and Xiong 2015). Rice is a critical cereal crop and is a primary focus for enhancing drought tolerance. However, its cultivation requires substantial water usage, rendering it particularly vulnerable to drought stress during the entire growth period (Todaka et al. 2015). Under natural circumstances, in the event that rice plants are deprived of sufficient water, the bulliform cells undergo a process of dehydration, which results in adaxially rolled leaves. In conditions where water is abundant, bulliform cells uptake water and undergo swelling, resulting in the leaves reverting back to their initial state (Price et al. 1997; Zou et al. 2024). Therefore, leaf rolling in rice caused by bulliform cells is significantly associated with drought stress. Through regulation on its target gene *HB4*, *miR166* controls the development of bulliform cells, and thus influence leaf morphology and drought tolerance (Zhang et al. 2018). Further investigation into the correlation between bulliform cells and drought stress is needed.

The homeodomain leucine zipper IV (HD-ZIP IV) family of transcription factors (TFs) is exclusive to the plant kingdom. The majority of HD-ZIP IVs are expressed in the outermost cells of plants and are known to be crucial players in several important developmental processes, such as trichome formation, epidermal cell differentiation, anthocyanin accumulation, root development, wax biosynthesis and bulliform cells development (Rerie et al. 1994; Lu et al. 1996; Kubo et al. 1999; Abe et al. 2003; Yu et al. 2008; Zou et al. 2011; Wang et al. 2018; Sun et al. 2020). Arabidopsis thaliana *GLABRA2* (*GL2*) is the first reported *HD-ZIP IV* gene, which is involved in the regulation of trichome development (Rerie et al. 1994). *Arabidopsis thaliana meristem L1* (*ATML1*) and *PROTODERMAL FACTOR2* (*PDF2*) play a critical role in differentiation and maintenance of epidermal cell fate (Lu et al. 1996; Abe et al. 2003). The *ANTHOCYANINLESS2* (*ANL2*) controls the anthocyanin pigmentation of the leaf subepidermal layer and cellular organization of the primary root (Kubo et al. 1999). *HOMEODOMAIN GLABROUS11* (*HDG11*) is capable of modulating the drought resistance of plants through the regulation of root architecture and leaf stomatal density (Yu et al. 2008). *HOMEODOMAIN GLABROUS2* (*HDG2*) is a key epidermal component promoting stomatal differentiation (Peterson et al. 2013). The rice genome contains nine genes homologous to *GL2*, known as *rice outermost cell-specific 1-9* (*ROC1-9*) (Ito et al. 2002, 2003). Among them, *ROC4* has been identified as a positive regulator of wax biosynthesis and drought tolerance (Wang et al. 2018). Both *ROC5* and *ROC8* have previously been found to exert a suppressive influence on bulliform cells development and participate in the regulation of leaf rolling (Zou et al. 2011; Sun et al. 2020). While ROC5 and ROC8 can individually form homodimers, they exhibit a greater inclination towards forming heterodimers. Neither the overexpression of *ROC8* in *ROC5* mutants, nor the overexpression of *ROC5* in *ROC8* knockout lines, can effectively alleviate the abaxially-rolled-leaf character. *ROC5* and *ROC8* demonstrate additive effects, with the abaxially rolled leaves being more pronounced in double mutants of *ROC5* and *ROC8* compared to single mutants of either genes (Xu et al. 2021). However, further investigation is necessary to ascertain the potential role of other *ROCs* in leaf rolling through participation in bulliform cells development. Further investigation into the correlation between *ROCs* and drought stress also deserves attention.

In this study, among the nine *ROC* genes encoding HD-ZIP IV family TFs in rice, *ROC1* exhibited the highest expression in the leaves. To study the function of *ROC1* in rice, we constructed transgenic plants with *ROC1* overexpression and knockout, and found that *ROC1* negatively regulated the development of bulliform cells and was involved in the regulation of rice leaf rolling. Furthermore, ROC1KO plants were drought sensitive, whereas ROC1OE plants responded to drought similar to the WT. Additionally, protein interaction assays revealed that ROC1 was capable of forming homodimers and heterodimers with ROC5 and ROC8 respectively. The double knockout of *ROC1* and *ROC8* plants not only exhibited abaxially rolled leaves due to increase in the size of bulliform cells, but also were more sensitive to drought than ROC1KO plants. However, *ROC8* was unable to restore the abaxially rolled leaves of ROC1KO plants. Thus, our results deepened the understanding of HD-ZIP IVs in regulation of leaf rolling and drought stress response through controlling bulliform cells development.

## Main Text

### Knockout of *ROC1 *Resulted in Abaxially Rolled Leaves, and Overexpression of *ROC1* Resulted in Adaxially Rolled Leaves

**Fig. 1:**
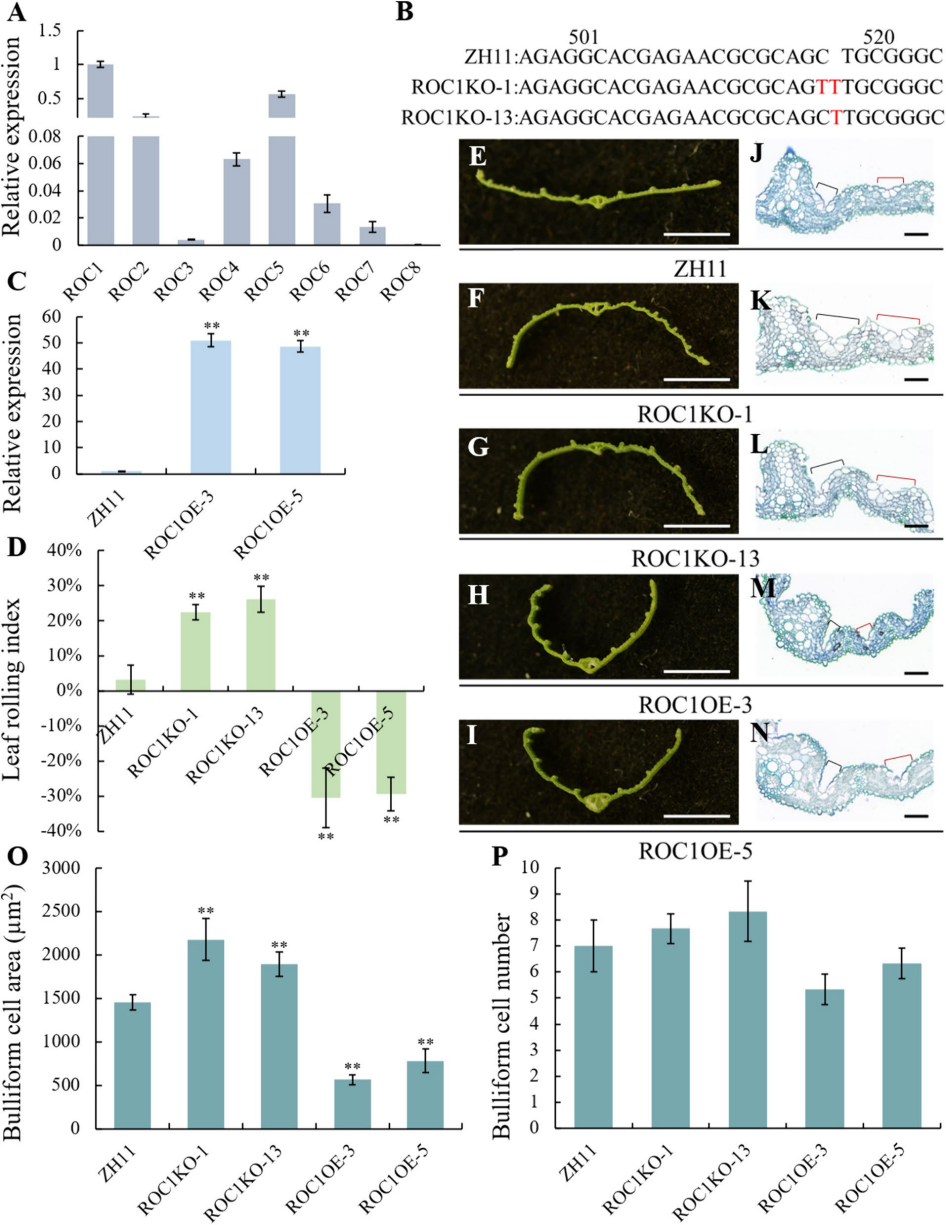
Phenotype analysis of the *ROC1* over expression and knockout lines. **A** The relative expression of ROC1-8 genes in 21-day-old seedling leaves of ZH11 (n = 3). **B** Sketch map of the edited sites (marked in red) in the ROC1KO-1 and ROC1KO-13 lines. In ROC1KO-1 line, a “T” was inserted and a “C” was replaced by a “T”, while in ROC1KO-13 line, a “T” was inserted in the corresponding ROC1 genomic region. **C** qRT-PCR assay of the expression of ROC1 gene in the ZH11, ROC1OE-3 and ROC1OE-5 plants (n = 3). **D** Leaf rolling index of ZH11, ROC1KO-1, ROC1KO-13, ROC1OE-3 and ROC1OE-5 flag leaves (n = 10). **E–I** Transverse sections of the flag leaves of ZH11, ROC1KO-1, ROC1KO-13, ROC1OE-3 and ROC1OE-5 plants (bar = 0.5 cm). **J**–**N** Transverse sections of the four-week-old seedling leaves of ZH11, ROC1KO-1, ROC1KO-13, ROC1OE-3 and ROC1OE-5 plants. Black brackets and red ones showing the bulliform cells adjacent to the main vein and the secondary vein respectively (bar = 50 μm). **O** The area of bulliform cells in the transverse section of leaves ofZH11, ROC1KO-1, ROC1KO-13, ROC1OE-3 and ROC1OE-5 plants (n = 3). **P** The number of bulliform cells in the transverse section of leaves ofZH11, ROC1KO-1, ROC1KO-13, ROC1OE-3 and ROC1OE-5 plants (n = 3). Bars represent the SD of measurements. Asterisks indicated significant differences compared with ZH11 plants as determined by Student’s *t*-test (**P < 0.01)

Both *ROC5* and *ROC8* have been reported to regulate leaf rolling by regulating the size of bulliform cells in rice (Zou et al. 2011; Sun et al. 2020). The overexpression of *ROC5* or *ROC8* causes a decrease in the size of bulliform cells, resulting in leaf rolling adaxially. Conversely, the knockout of *ROC5* or *ROC8* leads to an increase in the size of bulliform cells, causing leaves to roll abaxially (Zou et al. 2011; Sun et al. 2020). All the nine *ROCs* encode HD-ZIP IV family TFs, and their subcellular localization was initially investigated. The fusion proteins of ROC1-eGFP to ROC8-eGFP were found to co-localize with DAPI signals, indicating their localization in the nucleus (Fig. S1). To get an overall understanding of the HD-ZIP IV family in rice, we analyzed expression of *ROC1-8* genes in the 21-day-old seedling leaves and mature flag leaves of ZH11 using qRT-PCR, and found that they all expressed in the leaves with varied levels, and among them, *ROC1* exhibited the highest level of expression in both 21-day-old seedlings and mature flag leaves (Fig. 1A and Fig. S2). Therefore, *ROC1-8* might all involved in leaf development. Expression of *ROC9* in the leaf is too low to be detected (data now shown), which might indicate a function other from leaf development.

For genetic function analysis of *ROC1*, we generated two edited lines using Crisper-CAS9 technology, ROC1KO-1 and ROC1KO-13. In the ROC1KO-1 plants, a “C” was substituted with a “T” and an additional “T” was inserted. And in the ROC1KO-13 plants, a “T” was inserted into the *ROC1* gene (Fig. 1B). Both mutation types result in subsequent amino acid sequence changes and premature termination of translation (Fig. S3A). As a result, the flag leaves of both *ROC1* knockout lines displayed abaxially rolled leaf phenotype (Fig. 1E–G). Correspondingly, the leaf rolling index of the ROC1KO-1 and ROC1KO-13 was higher than that of wild type (WT) ZH11 (Fig. 1D). In the transversal section of the leaves, it was clear that the size of bulliform cells, whether adjacent to the main vein, or to the secondary vein in both lines of the ROC1KO plants were increased (Fig. 1J–L, O, P).

Meanwhile, we generated *ROC1* overexpression plants and got two lines with obvious up-regulation of *ROC1*, designated as ROC1OE-3 and ROC1OE-5 respectively (Fig. 1C). Leaves of both ROC1OE-3 and ROC1OE-5 lines rolled adaxially (Fig. 1E, H, I). Accordingly, it was revealed that the leaf rolling index of these two lines was much lower than that of ZH11 (Fig. 1D). Also, in the transversal section of the leaves, the size of bulliform cells, whether adjacent to the main vein, or to the secondary vein in the ROC1OE lines were decreased (Fig. 1J, M–P). Thus, these data collectively suggested that *ROC1* negatively regulated the development of bulliform cells, and thereby impacted leaf rolling.

### Knockout of *ROC1* Rendered Plants Drought Sensitive

**Fig. 2:**
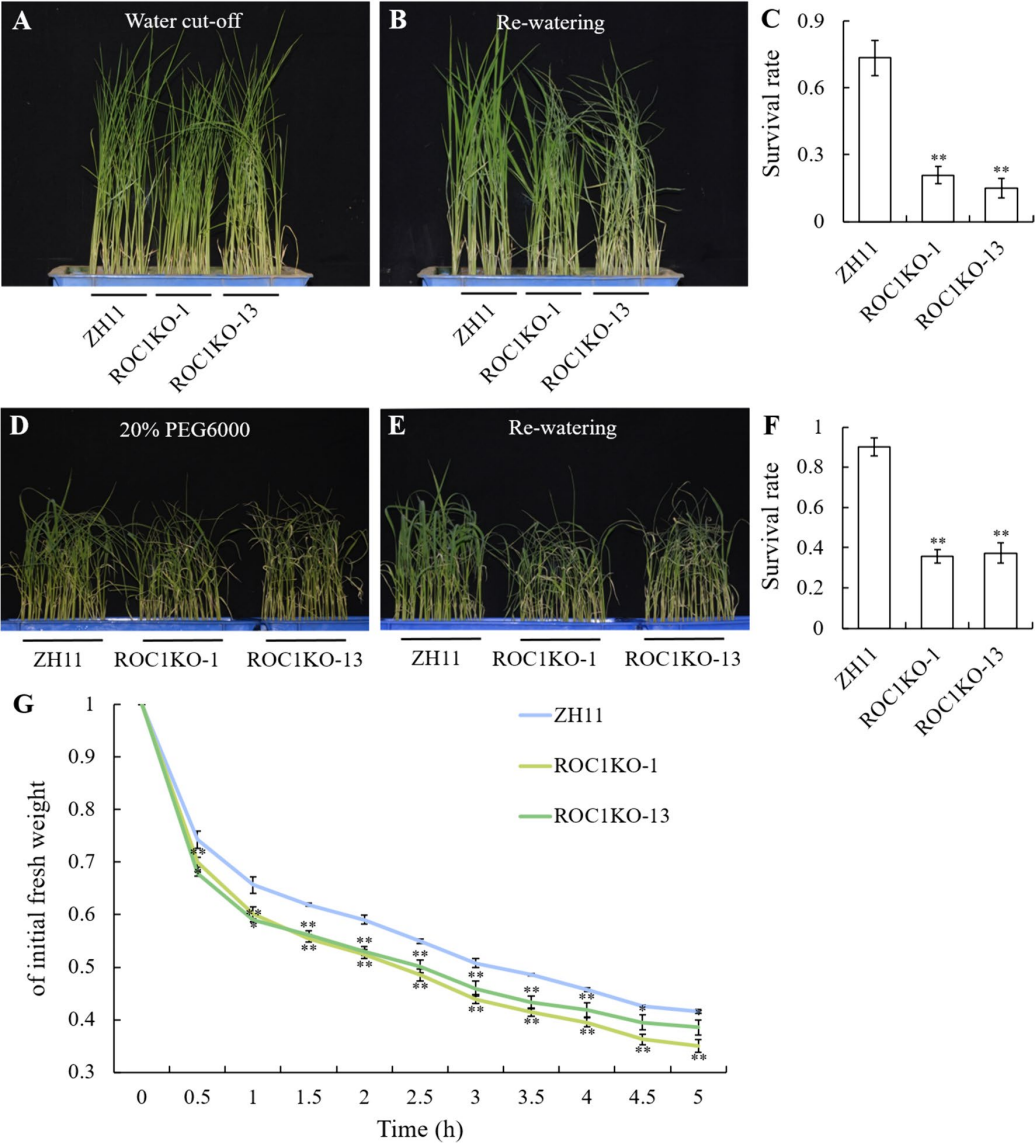
Drought tolerance detection of the ROC1KO plants and WT ZH11 plants. **A** The status of the ZH11, ROC1KO-1 and ROC1KO-13 plants after water cut-off treatment for 7 days. **B** The status of the ZH11, ROC1KO-1 and ROC1KO-13 plants after re-watering for 2 days. **C** The survival rates of the plants in **B**. **D** The status of the ZH11, ROC1KO-1 and ROC1KO-13 plants after 20% PEG6000 treatment for 9 days. **E** The status of the ZH11, ROC1KO-1 and ROC1KO-13 plants after re-watering for 2 days. **F** The survival rates of the plants in **E**. **G** The water losing rates of the leaves in ZH11, ROC1KO-1 and ROC1KO-13 plants. Bars represent the SD of measurements (n = 3). Asterisks indicated significant differences compared with ZH11 plants as determined by Student’s *t*-test (*P < 0.05, **P < 0.01)

In situations where water is lost, the bulliform cells experience a loss of water leading to shrinkage, which causes the leaves to roll adaxially (Zou et al. 2024). Bulliform cells could play a crucial role in enabling rice to adapt to drought conditions. To examine the association between *ROC1* and drought stress in rice, we employed three methods, direct water cut-off, 20% PEG6000 treatment to simulate a drought stress and water loss efficiency assay to assess the drought response of the ROC1KO, ROC1OE and WT ZH11 plants. In direct water cut-off, plants grown in plastic containers were cut-off watering at about three-leaf-stage (Fig. S4A). When the plants turned wilt (Fig. 2A), re-watering was applied to see the recovery of them (Fig. 2B). It was revealed that more plants in ROC1KO-1 and ROC1KO-13 lines died than ZH11 (Fig. 2B), accordingly, the survival rate of ROC1KO-1 and ROC1KO-13 plants was lower than that of ZH11 (Fig. 2C).

Similarly, in 20% PEG6000 treatment, the nutrient solution was replaced by that with 20% PEG6000 (Fig. S4B). Once the plants showed signs of wilting (Fig. 2D), they were returned to the nutrient solution again (Fig. 2E). The result was similar to that in direct water cut-off treatment, more ROC1KO-1 and ROC1KO-13 plants died than ZH11 plants (Fig. 2E), with the survival rates lower than that of ZH11 (Fig. 2F). We further measured the rate of water loss in ROC1KO-1, ROC1KO-13 and ZH11 plants, and found that the dry weight of ROC1KO-1 and ROC1KO-13 plants decreased more rapidly than that of ZH11 plants (Fig. 2G). Thus, the ROC1KO plants were sensitive to drought. In addition, no significant difference was revealed between ROC1OE-3 and ROC1OE-5 plants, and WT plants in neither direct water cut-off assay nor 20% PEG6000 treatment (Fig. S5).

### ROC1 Could Form Homodimers and Heterodimers with ROC5 and ROC8 Respectively

**Fig. 3:**
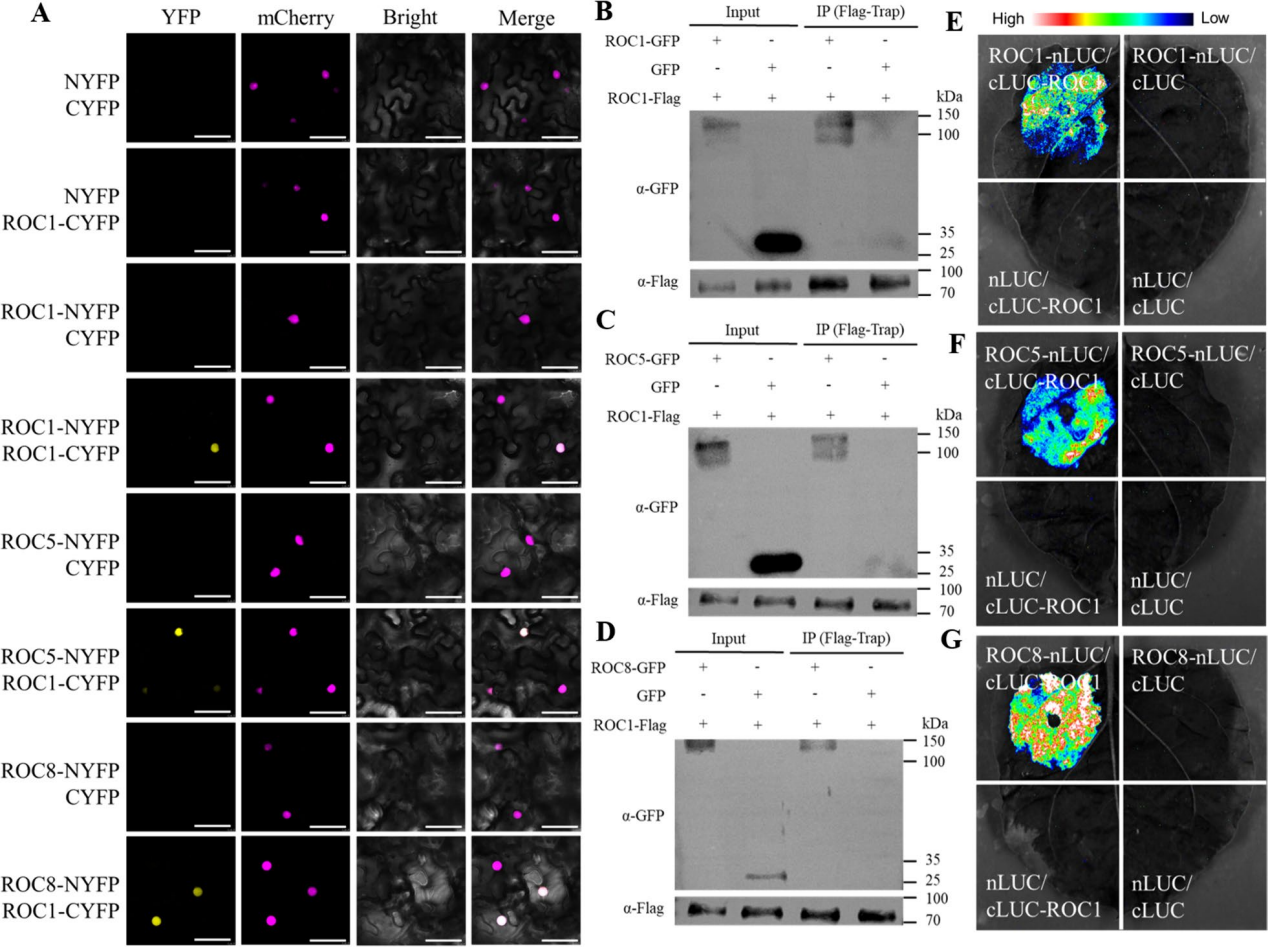
ROC1 interacts with ROC1, ROC5 and ROC8 proteins verified by BiFC, Co-IP and LCI assays. **A** BiFC assay to detect the interaction between ROC1 and ROC1, ROC5, and ROC8 proteins as indicated respectively (bar = 50 µm). H2B-mCherry protein was used as a nuclear marker. **B** Co-IP assay to detect the interaction between ROC1 and ROC1 proteins. **C** Co-IP assay to detect the interaction between ROC1 and ROC5 proteins. **D** Co-IP assay to detect the interaction between ROC1 and ROC8 proteins. **E** LCI assay to detect the interaction between ROC1 and ROC1 proteins. **F** LCI assay to detect the interaction between ROC1 and ROC5 proteins. **G** LCI assay to detect the interaction between ROC1 and ROC8 proteins

The HD-ZIP IV family TFs function through formation of homodimers and heterodimers (Ito et al. 2003). We detected the corresponding character of ROC1. By conducting bimolecular fluorescence complementation (BiFC) experiments in tobacco leaves, we showed that ROC1-NYFP can interact with ROC1-CYFP, ROC5-CYFP and ROC8-CYFP within the nucleus, resulting in the production of an YFP fluorescence signal (Fig. 3A). In the co-immunoprecipitation (Co-IP) experiments, we co-expressed ROC1-Flag together with ROC1-GFP, ROC5-GFP and ROC8-GFP proteins respectively. The samples were then enriched using the Flag-Trap method and subjected to Western blot assay using GFP antibodies. The presence of ROC1-GFP, ROC5-GFP and ROC8-GFP was verified, while the control GFP was absent (Fig. 3B–D). Thus, the interaction between ROC1-Flag and ROC1-GFP, ROC5-GFP and ROC8-GFP was verified. Additionally, in the luciferase complementation imaging assay (LCI) experiments, cLUC-ROC1 interacted with ROC1-nLUC, ROC5-nLUC and ROC8-nLUC, all displaying strong LUC fluorescence signals (Fig. 3E–G). Collectively, these data confirmed that ROC1 could form homodimers via self-association and heterodimers with ROC5 and ROC8 respectively.

### ROC1 Demonstrated a Higher Inclination for Forming Heterodimers with ROC8

**Fig. 4:**
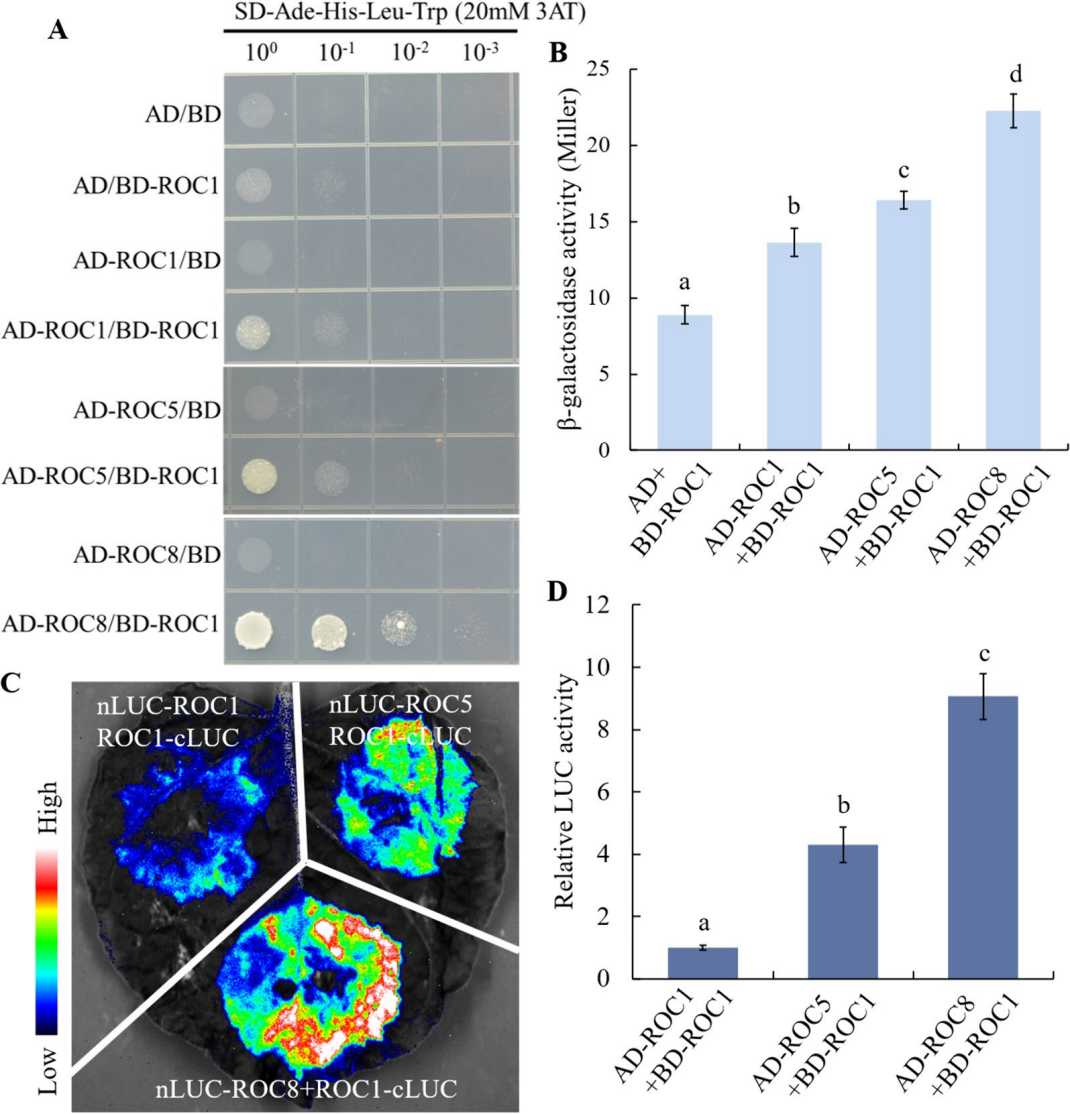
Detection of the interaction intensity of ROC1 with ROC1, ROC5 and ROC8 proteins. **A** Y2H assay to detect the interaction intensity of ROC1 and ROC1, ROC1 and ROC5, and ROC1 and ROC8 proteins as indicated respectively. 20 mM 3AT was used to inhibit the transcriptional activation activity of ROC1 and the expression of the HIS3 reporter gene. **B** β‐galactosidase activity assay to detect the interaction intensity of ROC1 and ROC1, ROC1 and ROC5, and ROC1 and ROC8 proteins as indicated respectively. **C** LCI assay to detect the interaction intensity of ROC1 and ROC1, ROC1 and ROC5, and ROC1 and ROC8 proteins. **D** Relative LUC activity in **C**. Bars represented the SD of measurements (n = 5). Letters indicated significant differences determined by one-way ANOVA with Tukey’s test (*P* < 0.01)

In previous reports, it was observed that ROC5 and ROC8 exhibited a higher tendency towards generating heterodimers (Xu et al. 2021). Here we used yeast two hybrid to check if ROC1 has a higher tendency to form heterodimers with ROC5 and ROC8. It turned out that the growth rate of yeast cells transformed with BD-ROC1 and AD-ROC8 was notably higher than that with other combinations. And the growth rate of yeast cells transformed with BD-ROC1 and AD-ROC5 was observed to be higher than that transformed with BD-ROC1 and AD-ROC1 respectively (Fig. 4A and Fig. S6). Subsequently, the yeast cells that had been successfully transformed with BD-ROC1 and AD, AD-ROC1, AD-ROC5 and AD-ROC8 were cultured at 30 °C for 2 days for the detection of β-galactosidase activity. It was revealed that the β-galactosidase activity of the yeast cells transformed with BD-ROC1 and AD-ROC8 was notably higher than that of the other combinations. Additionally, the activity of β-galactosidase of the yeast cells transferred with BD-ROC1 and AD-ROC5 was significantly higher than those transferred by BD-ROC1 and AD-ROC1 respectively (Fig. 4B). Furthermore, ROC1-cLUC was injected with nLUC-ROC1, nLUC-ROC5, and nLUC-ROC8 respectively into the same *N. benthamiana* leaves. The ROC1-cLUC and nLUC-ROC8 clones displayed significantly stronger LUC fluorescence compared to other combinations. Moreover, ROC1-cLUC and nLUC-ROC5 also exhibited markedly higher LUC fluorescence than ROC1-cLUC and nLUC-ROC1 combination (Fig. 4C), in accordance with the quantification of LUC/REN strength (Fig. 4D). Altogether, ROC1 had greater inclination towards formation of heterodimers with ROC8, and the inclination of forming heterodimer with ROC5 was also higher than forming homodimer.

### Simultaneous Knockout of *ROC1 *and *ROC8* Resulted in More Severe Leaf Abaxial Rolling and More Sensitive to Drought

**Fig. 5:**
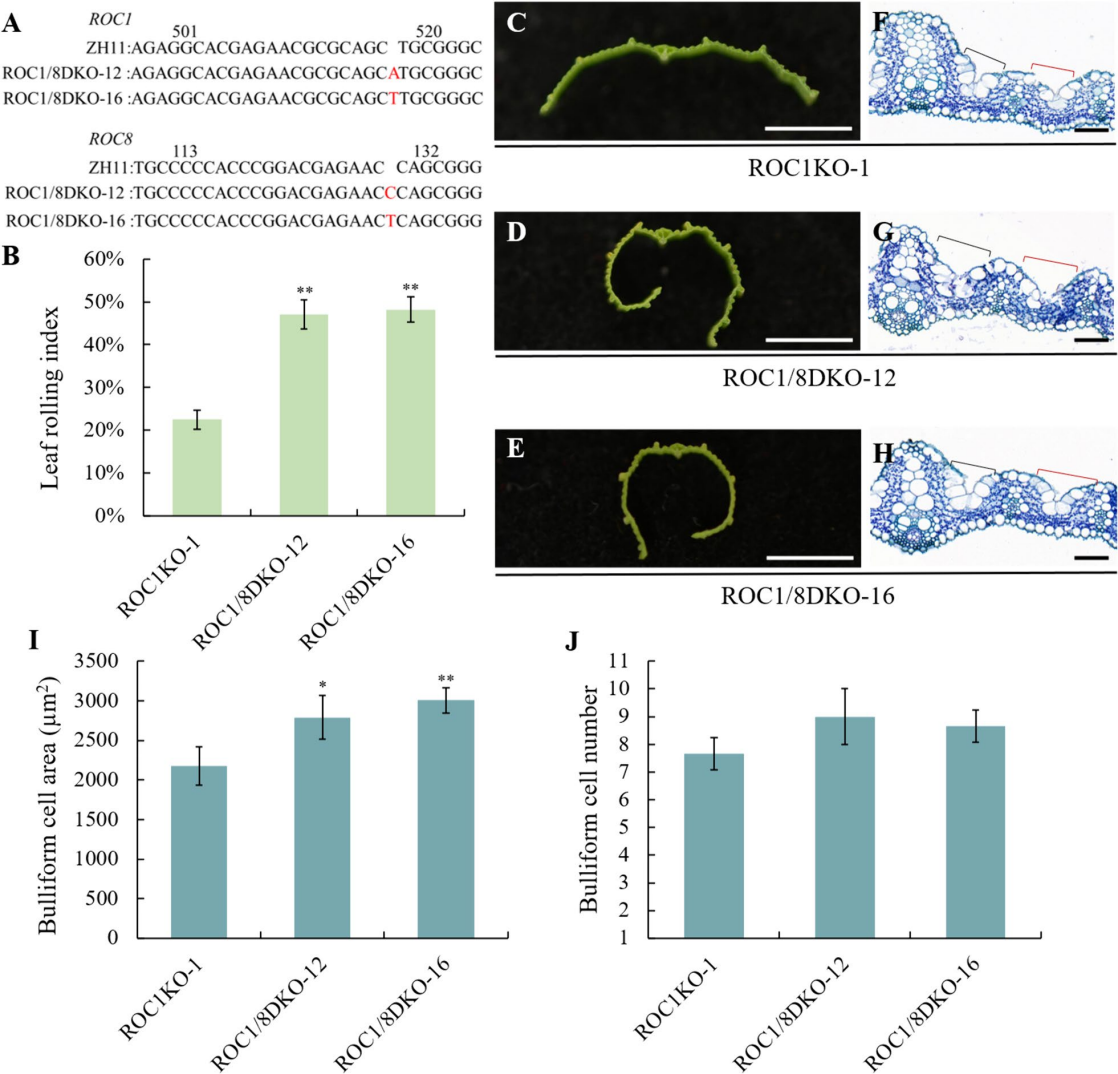
Leafing rolling intensity of ROC1 and ROC8 double knocked out plants in comparison with ROC1KO-1 plants. **A** Sketch map of the edited sites in the ROC1/8DKO-12 and ROC1/8DKO-16 plants. In ROC1/8DKO-12 plants, an “A” was inserted in the ROC1 gene, and a “C” was inserted in the ROC8 gene. In ROC1/8DKO-16 plants, a “T” was inserted in the ROC1 gene, and a “T” was inserted in the ROC8 gene. **B** Leaf rolling index of ROC1KO-1, ROC1/8DKO-12 and ROC1/8DKO-16 flag leaves (n = 10). **C**–**E** Transverse sections of the flag leaves of ROC1KO-1, ROC1/8DKO-12 and ROC1/8DKO-16 plants (bar = 0.5 cm). **F**–**H** Transverse sections of the four-week-old seedling leaves of the ROC1KO-1, ROC1/8DKO-12 and ROC1/8DKO-16 plants. Black brackets and red ones showing the bulliform cells adjacent to the main vein and the secondary vein respectively (bar = 50 μm). **I** The area of bulliform cells in the section of leaves of ROC1KO-1, ROC1/8DKO-12 and ROC1/8DKO-16 plants (n = 3). **J** the number of bulliform cells in the section of leaves of ROC1KO-1, ROC1/8DKO-12 and ROC1/8DKO-16 plants (n = 3). Bars represented the SD of measurements. Asterisks indicated significant differences compared with ROC1KO-1 plants as determined by Student’s *t*-test (*P < 0.05, **P < 0.01)

Since *ROC1*, like *ROC8* (Sun et al. 2020), negatively regulated development of bulliform cells to affect leaf rolling (Fig. 1D–N), and ROC1 showed higher inclination to interact with ROC8 (Fig. 4), we wonder if *ROC1* and *ROC8* have additive effect on leaf rolling. To this end, we constructed two lines with *ROC1* and *ROC8* double knockout using Crisper-CAS9 technology, namely ROC1/8DKO-12 and ROC1/8DKO-16. In ROC1/8DKO-12 plants, an “A” was inserted into the *ROC1* gene and a “C” was inserted into the *ROC8* gene. In ROC1/8DKO-16 plants, a “T” was inserted into the *ROC1* gene and a “T” was inserted into the *ROC8* gene (Fig. 5A). Each of these mutation types results in subsequent amino acid sequence changes and premature termination of translation (Fig. S3). As a result, the degree of the leaf abaxial rolling of both lines of ROC1/8DKO plants were significantly higher than that of ROC1KO plants, as manifested in the transverse section (Fig. 5C–E), and the rolling index measurement (Fig. 5B). In the transversal section of the leaves, it was clear that the size of bulliform cells, whether adjacent to the main vein, or adjacent to the secondary vein in ROC1/8DKO plants were all increased than that in ROC1KO plants (Fig. 5F–J). These results supported the notion that *ROC1* and *ROC8* have an additive effect when it comes to negatively regulating rice bulliform cells development and influencing leaf rolling.

**Fig. 6:**
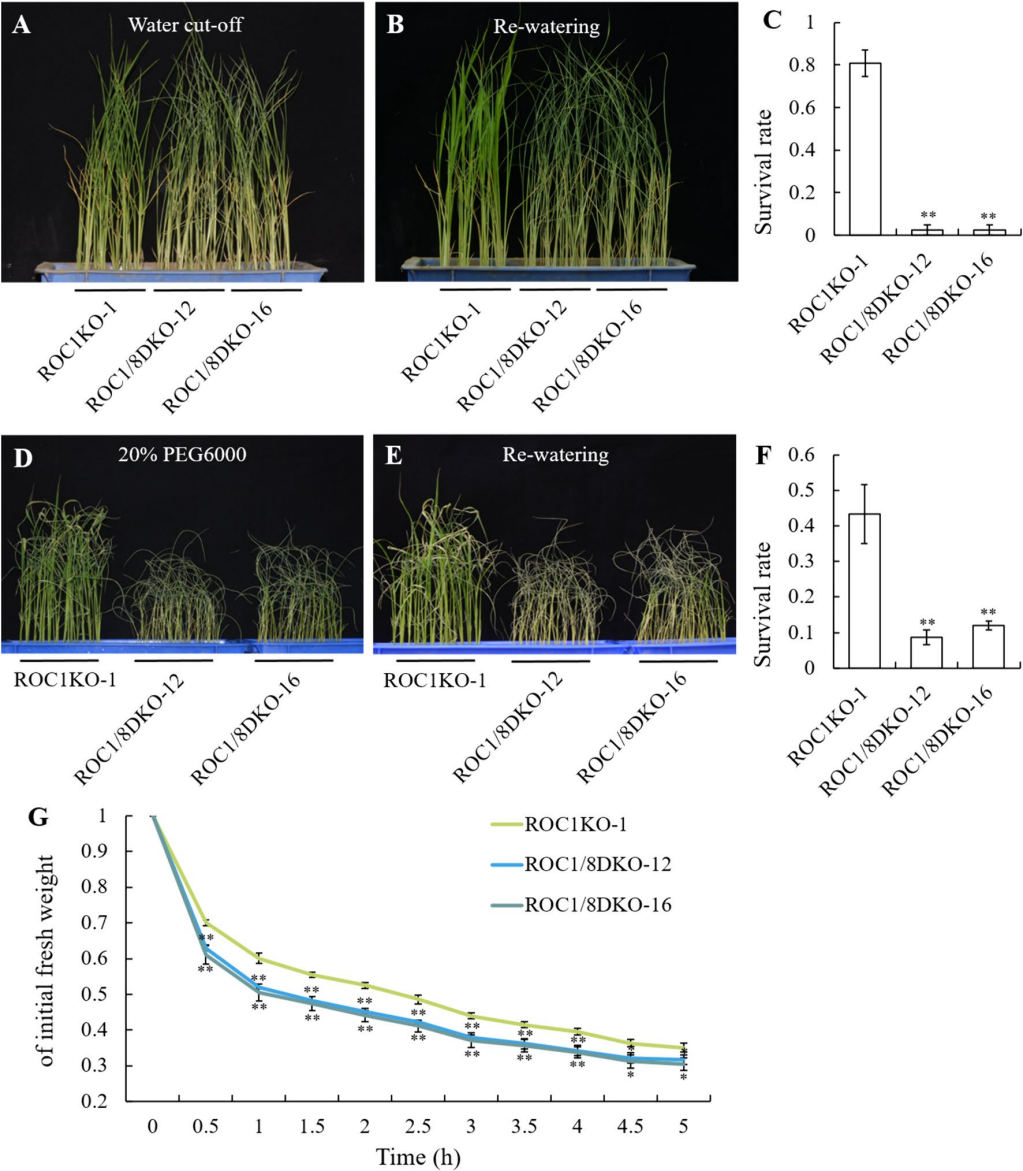
Drought tolerance detection of ROC1 and ROC8 double knocked out plants and ROC1KO-1 plants. **A** The status of the ROC1KO-1, ROC1/8DKO-12 and ROC1/8DKO-16 plants after water cut-off treatment for 6 days. **B** The status of the ROC1KO-1, ROC1/8DKO-12 and ROC1/8DKO-16 plants after re-watering for 2 days. **C** The survival rates of the plants in **B**. **D** The status of the ROC1KO-1, ROC1/8DKO-12 and ROC1/8DKO-16 plants after 20% PEG6000 treatment for 9 days. **E** The status of the ROC1KO-1, ROC1/8DKO-12 and ROC1/8DKO-16 plants after recovery for 2 days. **F** The survival rates of the plants in **E**. **G** The water losing rates of the leaves of ROC1KO-1, ROC1/8DKO-12 and ROC1/8DKO-16 plants. Bars represented the SD of measurements (n = 3). Asterisks indicated significant differences compared with ROC1KO-1 plants as determined by Student’s *t*-test (*P < 0.05, **P < 0.01)

Since knockout of *ROC1* leads to an increased susceptibility of plants to drought stress (Fig. 2), it is hypothesized that a double knockout of both *ROC1* and *ROC8* may lead to a further increased plant susceptibility to drought stress. For verification, the ROC1/8DKO-12 and ROC1/8DKO-16 plants were subjected to direct water cut-off and 20% PEG6000 treatment together with ROC1KO plants. In direct water cut-off, the majority of ROC1/8DKO-12 and ROC1/8DKO-16 plants died, while a large number of ROC1KO-1 plants survived (Fig. S4C, 6A, B), accordingly, the survival rate of ROC1/8DKO-12 and ROC1/8DKO-16 plants were significantly lower than that of ROC1KO-1 plants (Fig. 6C). Similarly, in 20% PEG6000 treatment, more ROC1/8DKO-12 and ROC1/8DKO-16 plants died than ROC1KO-1 plants (Fig. S4D, 6D, E), with the survival rate of ROC1/8DKO plants lower than that of ROC1KO-1 plants (Fig. 6F). Additionally, the rate of water loss was assessed in ROC1/8DKO-12, ROC1/8DKO-16, and ROC1KO-1 plants. It was observed that the dry weight of ROC1/8DKO-12 and ROC1/8DKO-16 plants declined at a faster rate than that of ROC1KO-1 plants (Fig. 6G). These findings suggested that the double knockout of *ROC1* and *ROC8* may lead to a further increased plant susceptibility to drought stress.

### Overexpression of *ROC8* in the ROC1KO-1 Plants Did Not Rescue the Abaxially Rolled Leaf Phenotype

**Fig. 7:**
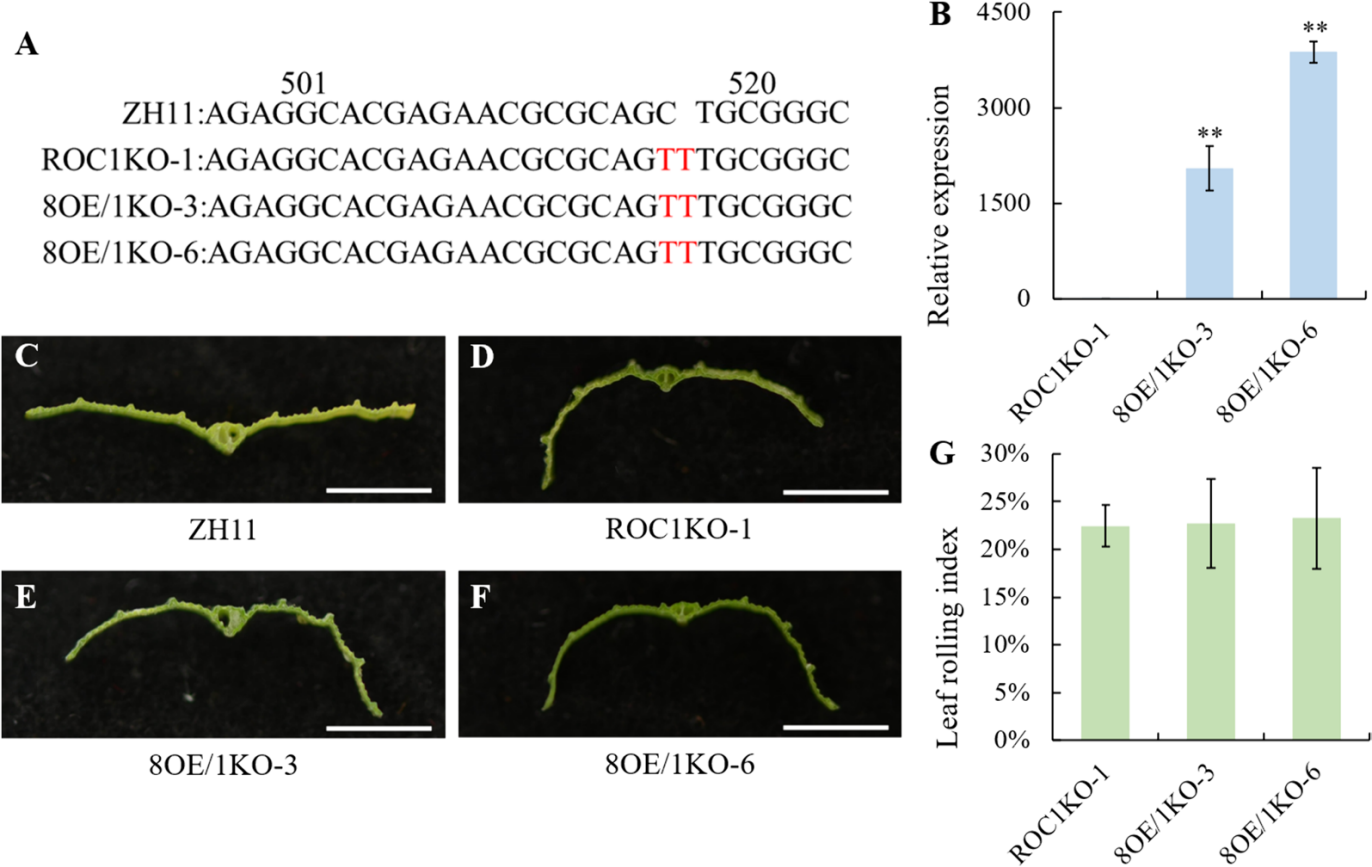
Characterization of overexpression of ROC8 in the ROC1KO-1 plants. **A** Sketch map of the edited sites in the 8OE/1KO-3, 8OE/1KO-6, and ROC1KO-1 plants. All plants inserted in a “T” and a “C” was replaced by a “T” in the corresponding ROC1 genomic region. **B** qRT-PCR assay of the expression of *ROC8* gene in the ROC1KO-1, 8OE/1KO-3 and 8OE/1KO-6 plants (n = 3). **C–F** Transverse sections of the ZH11, ROC1KO-1, 8OE/1KO-3 and 8OE/1KO-6 flag leaves as indicated respectively (bar = 0.5 cm). **G** Leaf rolling index of the ROC1KO-1, 8OE/1KO-3 and 8OE/1KO-6 flag leaves (n = 10). Bars represented the SD of measurements. Asterisks indicated significant differences compared with ROC1KO-1 plants as determined by Student’s *t*-test (**P < 0.01)

To further explore the regulatory role of *ROC1* and *ROC8* in rice leaf rolling, we overexpressed *ROC8* in ROC1KO-1 plants. Specifically, we generated 8OE/1KO-3 and 8OE/1KO-6 plants where the *ROC1* gene was knocked out as in ROC1KO-1 (Fig. 7A), and *ROC8* was over expressed (Fig. 7B). Remarkably, the flag leaves of both 8OE/1KO-3 and 8OE/1KO-6 plants were abaxially rolled, to a degree similar to that of ROC1KO-1 plants, which was obvious from the transverse section (Fig. 7C–F), and the rolling index measurement (Fig. 7G). Thus, there may be some additive effects on the functions of *ROC1* and *ROC8*, but the specific regulatory functions may still depend on their individual properties.

## Discussion

The epidermis is the outermost cell layer of plants, and epidermal cells are specially differentiated that vary in size, morphology, and function (Guimil and Dunand 2007). Epidermal cells undergo a series of intricate regulatory processes, leading to the development of diverse cell types including bulliform cells, stomata, trichomes, and others (Zou et al. 2011; Vernoud et al. 2009; Takada and Iida 2014). The bulliform cells constitute the predominant determinant in the modulation of rice leaf rolling, with their atypical differentiation resulting in aberrant leaf rolling (Li et al. 2010; Hong et al. 2002). Currently, a few genes have been identified to be associated with the regulation of bulliform cells development in rice (Zhao et al. 2010; Hu et al. 2010; Hibara et al. 2009). The TFs ROC5 and ROC8 have a negative impact on the development of bulliform cells, and consequently influencing leaf rolling (Zou et al. 2011; Sun et al. 2020). The ROC family TFs is named by their fundamental roles in epidermal regulation (Ito et al. 2002, 2003). In this study, we revealed that, *ROC1*, another gene encoding HD-ZIP IV transcription factor in the ROC family, also negatively regulated development of bulliform cells, with alteration in expression resulting in leaf rolling (Fig. 1D–N). Investigating the role of ROC family TFs in regulating bulliform cells and leaf rolling in rice holds significant scientific value.

Sharing the same conserved domain (Ito et al. 2003), the ROC family TFs specify the character of epidermal cells, and specifically in rice, they might associated tightly with bulliform cell development as it stands. *ROC5* was initially identified in a T-DNA insertion mutant called *outcurved leaf1* (*oul1*), which exhibited a distinctive abaxially rolled leaves that was attributable to an elevated number and enlarged size of bulliform cells. Subsequently, the leaves of *ROC5* overexpressing plants displayed a characteristic adaxially rolled, accompanied by a decrease in both the number and size of bulliform cells (Zou et al. 2011). *ROC8* was discovered in the *crm1-D* mutant that displays adaxially rolled leaves, accompanied by an increase in the size of bulliform cells (Sun et al. 2020). *ROC1* functions similarly with *ROC5* and *ROC8* in negative regulation on rice bulliform cells and influencing leaf rolling (Fig. 1D–P). The *ROC1* exhibited the highest level of expression in both 21-day-old seedlings and mature flag leaves, followed by *ROC5*, while *ROC8* displayed notably low expression (Fig. 1A, Fig. S2). Therefore, perturbations in the expression of *ROC1*, *ROC5* and *ROC8* can lead to alterations in the development of bulliform cells, and ultimately result in leaf rolling. Notably, the expression levels of *ROCs* may not necessarily correlate with its regulatory efficacy.

Members of ROC family TFs form homodimers or heterodimers associations for their functional activities (Xu et al. 2021; Ito et al. 2003). Both ROC5 and ROC8 can form homodimers on their own, and meanwhile form heterodimers with each other. Furthermore, ROC5 and ROC8 are more inclined to form heterodimers to exert their functions (Xu et al. 2021).Our findings indicated that ROC1 formed homodimers as well as heterodimers with ROC5 and ROC8 respectively (Fig. 3). Nonetheless, the interaction between ROC1 and ROC8 was the strongest, and that between ROC1 and ROC5 went the next, whereas the interaction intensity for ROC1 self-assembly into a homodimer was relatively weak (Fig. 4). In comparison to *ROC1* single-knockout plants, *ROC1* and *ROC8* double-knockout plants not only exhibited higher degree of abaxially rolled leaves due to the increase in the size of bulliform cells (Fig. 5), but also further increased plant sensitivity to drought stress (Fig. 6). Nevertheless, the overexpression of *ROC8* does not lead to the restoration of abaxially rolled leaves in ROC1KO plants (Fig. 7), indicating that the function of *ROC1* may not depend on ROC8. Considering similar result of ROC5 and ROC8, it is highly possible that different members of the ROC family TFs in rice tend to form heterodimers, but the specific regulatory functions may still mainly depend on themselves. Elucidation of function of other ROC members would further verify this possibility.

As a physiological indicator, the bulliform cells could sense the environmental water status and swell or shrink accordingly, and thus result in the bending or stretching of rice leaves. In water-deficient conditions, the bulliform cells shrink and lead to adaxially rolled leaves. This physiological process is crucial for rice to withstand water loss. Upon rehydration, the bulliform cells reabsorb water and expand, allowing the leaves to return to their original flat state (Price et al. 1997; Zou et al. 2024). ROC1KO plants led to an increase in the size of bulliform cells (Fig. 1J–L, O), and thus resulted in abaxially rolled leaves (Fig. 1D–G). In response to drought, the leaves of the ROC1KO plants rolled more quickly and accordingly the ROC1KO plants exhibited a drought-sensitive phenotype (Fig. 2), this hypersensitivity to water deficiency might be attributed to the increased bulliform cells. While ROC1OE plants were similar to WT in response to drought (Fig. S5). The bulliform cells in the *dynamic leaf rolling 1* (*dlr1*) mutant are even more sensitive to water deficiency, which could shrink during noon when water is deficient due to the high temperature and vigorous transpiration, and accordingly the leaves roll inwardly at this point. But the leaves turn normal in the morning and afternoon when water deficiency is relieved (Zou et al. 2024). In accordance with this hypothesis, the simultaneous knockout of both *ROC1* and *ROC8* genes exacerbated abaxially rolled leaves (Fig. 5) and further increased the plant's susceptibility to drought (Fig. 6). Moreover, the formation of stomata in rice is also influenced by the intricate development of epidermal cells, which play a role in controlling water loss through dispersion. Our scanning electron microscopy (SEM) analysis revealed that ROC1 does not participate in the regulation of stomatal development in rice (Fig. S7). Therefore, we identified a *ROC1* gene that could regulate bulliform development and drought tolerance in rice, which provide some molecular basis for the function of bulliform cells in mediating water homeostasis. In future research, some more detailed molecular mechanism on the direct connection between the development or state of bulliform cells and drought tolerance might still be needed.

## Conclusions

Our findings revealed that the HD-ZIP IV family gene *ROC1* negatively regulated the development of bulliform cells in rice, with over expression of *ROC1* resulting in decreased size of bulliform cells and adaxial leaf rolling, while knocking out of *ROC1* resulting in increased size of bulliform cells and abaxial leaf rolling respectively. Meanwhile, knockout of *ROC1* rendered plants sensitive to drought. Regulation of *ROC1* on the development of bulliform cells and leaf rolling was probably achieved through formation of heterodimers with ROC5 and ROC8. In comparison with ROC1KO plants, ROC1/8DKO plants not only showed a greater degree of abaxial leaf rolling due to increase the size of bulliform cells, but also demonstrated even greater susceptibility to drought stress. Function of *ROC1* and *ROC8* might have additive effect. However, the precise regulatory role of *ROC1* may also rely on its individual properties. This study revealed the function of *ROC1* in the regulation of leaf rolling and drought stress response through modulating bulliform cells development, a potential close relationship among different ROC members in their biological function was indicated.

## Material and Methods

### Plant Species and Growth Conditions

The wild type (WT) rice plants used in this study were varieties ZH11 (*Oryza sativa* L. subsp. *japonica* cv. Zhonghua No.11, ZH11). ZH11 was mainly used as host of genetic transformation or natural WT of mutants. All rice plants were cultivated under field conditions at experimental stations in Shanghai (30°N, 121°E), China. Rice seedlings were cultures in the phytotron in CAS Center for Excellence in Molecular Plant Sciences, with 30/24 ± 1 °C day/night temperature, 50–70% relative humidity and a light/dark period of 14 h/10 h was used to culture rice seedlings.

### Plasmid Construction and Plant Transformation

For over expression of *ROC1*, full length cDNA of *ROC1* was amplified and cloned into p1301-35S-Nos vector through digestion by *Xba*I and *Kpn*I.

For over expression of *ROC8* in the ROC1KO-1 background, full length cDNA of *ROC8* was amplified and cloned into the pCAMBIA2301 vector through digestion by *Xba*I and *Kpn*I.

For construction of knock-out plants of ROC1KO and ROC1/8DKO, guider DNA was respectively synthesized and cloned in the pOs-sgRNA vector, and then transferred to the pH-Ubi-cas9-7 vector through LR reaction. Primers and gDNAs used were listed in Table S1.

Plasmids were respectively transformed into ZH11 or ROC1KO-1 through *Agrobacterium*-mediated genetic transformation in Biorun Biosciences Company.

### RNA Isolation and Quantitative Real–Time RT–PCR (qRT–PCR) Analysis

Total RNAs were extracted using TRIzol (Life technologies, USA) and reverse transcribed using the First Strand cDNA Synthesis Kit (Toyobo). qRT–PCR was performed with the SYBR Green Real–time PCR Master Mix Kit (Toyobo) with a reaction system containing 10 μl of 2× SYBR Green Real-time PCR Master Mix, 0.4 μl of each primer, 4 μl of 10 ng/μl cDNA, and 5.2 μl of ddH2O. qRT-PCR was conducted using an Eppendorf realplex2 System. The rice Actin (LOC_Os03g50885) and Ubiquitin (LOC_Os01g22490) were used as reference genes to normalize expression levels. Relative gene expression in different individuals was analyzed using the 2−ΔΔCT method with Actin, compared with wild type (Livak and Schmittgen 2001). Methods for analyzing qPCR with two reference genes was according to the website (https://toptipbio.com/qpcr-multiple-reference-genes/). Data from three biological repeats were collected, and the mean value with standard deviation was plotted.

All the primer sequences used in qRT–PCR and other analysis in this study were listed in Table S1.

### Measurement of Leaf Rolling Index (LRI)

LRI was measured as previously described (Shi et al. 2007) and calculated as follows: LRI (%) = (Lw – Ln)/Lw × 100. Lw: expand the leaf blade and determine the greatest width of the leaf blade. Ln: at the same site, measure the natural distance of the leaf blade margins. Data were measured from the middle of the flag leaf of 10 individual plants in the field.

### Anatomical Analysis

The middle parts of the third leaf of four-week-old rice were soaked in 50% FAA (50% anhydrous ethanol, 5% glacial acetic acid, 5% formaldehyde) and vacuumed. After continuous dehydration in different concentrations of ethanol, the samples were transferred to xylene and then embedded in paraffin. Then samples were cut into 8 μm sections and placed on a hot plate at 42 °C overnight. Sections were stained with 0.5% toluidine blue O for 30 min at 37 °C, washed with water and dried at 37 °C. After paraffin removal using xylene, the sections were sealed and photographed under a microscope. The number and size of bulliform cells were measured by Image J software for bulliform cells adjacent to the main vein at the same magnification. Data from three biological repeats were collected, and the mean value with standard deviation was plotted.

For manual sections, middle parts of freshly collected flag leaves were manually cut into about 0.5 mm slices as quickly as possible, and pictures were taken under NIKON D7000.

### Drought Tolerance Detection

Drought tolerance was carried out using direct water cut-off and 20% PEG6000 treatment. For direct water cut-off, sprouted seeds of the tested and control plants (approximately 40 plants for each group) were planted into paddy soil side by side in a 13 × 18 cm plastic box, and grew in the phytotron for 4 weeks, after which watering was ceased until the plants wilted. Followed by re-watering for 2 days to observe their recovery status.

For 20% PEG6000 treatment, sprouted seeds were planted in a plastic plate with 3 L rice nutrient solution (Yoshida) in the phytotron for 3 weeks, with the solution being changed every 2 days. Using the rice nutrient solution as a solvent, 3 L of 20% (w/v) PEG6000 solution was prepared. Subsequently, the entire rice nutrient solution in the plastic plate was replaced with 20% (w/v) PEG6000 solution until the plants wilted. Then, they were switched back to the rice nutrient solution for 2 days to observe their recovery status.

The survival rate was calculated from those that had recovered from leaf shrinkage and wilting with the following formula, (the number of surviving plants / the total number of plants) × 100%.

### Calculation of Water Loss Efficiency

The water loss efficiency assay was performed as described (Tian et al. 2015). The 3-week-old rice seedlings were cut from the base and weighed with a microbalance at 0, 0.5, 1, 1.5, 2, 2.5, 3, 3.5, 4, 4.5 and 5 h. A time-course analysis was conducted to examine the rate of water loss, which was expressed as a percentage of the initial fresh weight at different time intervals.

### Bimolecular Fluorescence Complementation (BiFC)

The cDNA of the coding region of *ROC1* was cloned into the *Kpn*I and *Sal*I sites of pCAMBIA1300-35S-cYFP using homologous recombination. Similarly, the cDNA sequences of the coding region of *ROC1*, *ROC5* and *ROC8* were cloned into the *Kpn*I and *Sal*I sites of pCAMBIA1300-35S-nYFP using homologous recombination. The recombinant and control vectors were transformed into *Agrobacterium* GV3101 respectively. Agrobacterium cells were re-suspended in infection solution (10 mM MES, 10 mM MgCl_2_ and 200 μM acetosyringone) at OD600 = 1.0. The prepared suspensions were infiltrated into *N. benthamiana* leaves. After culture for 2 days, the signals of YFP and mCherry were detected using confocal microscopy (Leica TSC SP8 STED 3X).

### Co-Immunoprecipitation Assay (Co-IP)

The full-length cDNA of *ROC1* was fused with FLAG tag, while the full-length cDNA of *ROC1*, *ROC5* and *ROC8* were respectively fused with the GFP tag. The fusion proteins were transiently expressed in *N. benthamiana* as described above. *N.* *benthamiana* leaves were harvested after 3 d and frozen in liquid nitrogen. Soluble proteins were extracted with NB1 buffer (50 mM Tris-MES (pH8.0), 500 mM sucrose, 1 mM MgCl_2_, 10 mM EDTA, 5 mM DTT, 1 mM PMSF, Cocktail). Immunoprecipitation was performed with anti-FLAG-affinity beads (Sigma-Aldrich). Lysates were incubated with the prewashed beads for 3 h. Then the beads were washed 6 times and solubilized in an appropriate volume of extraction buffer with 1 × SDS loading buffer. The fusion proteins were detected by immunoblotting using monoclonal anti-FLAG M2 antibody (Sigma-Aldrich) and monoclonal antibody anti-GFP (Huabio). Western blot was performed according to the procedures described previously (Zhang et al. 2019).

### Luciferase Complementation Imaging Assay (LCI)

The full-length cDNA of the *ROC1* gene was cloned into the *Kpn*I and *Sal*I sites of pCAMBIA1300-CLuc using homologous recombination. While the full-length cDNA of *ROC1*, *ROC5* and *ROC8* coding sequences were respectively cloned into the *Kpn*I and *Sal*I sites of pCAMBIA1300-NLuc using homologous recombination. The fusion proteins were transiently expressed in *N. benthamiana* as described above. At 2 days post infiltration, the LUC signal was detected using a CCD camera and a 150 mg/mL luciferin solution.

In the protein–protein interaction intensity assay, the empty vector pGreenII 0800-LUC and recombinant vectors were co-transformed into *N. benthamiana*. After culture for 2 days, the signal of LUC was detected by CCD. The double luciferase reporter gene analysis system (Promega, Madison, WI) was used to quantitatively detect the enzyme activity of LUC and REN. Five biological replicates were set for each sample.

### Yeast Two-Hybrid Assay (Y2H)

The cDNA sequence of the encoding region of *ROC1* was constructed into the *BamH*I site of pGBKT7 using homologous recombination to get the BD-ROC1. The cDNA sequences of the coding region of *ROC1*, *ROC5* and *ROC8* were respectively constructed into the *BamH*I site of pGADT7 using homologous recombination to get AD-ROC1, AD-ROC5 and AD-ROC8. The constructed vectors were transformed into AH109 strain after pairing using a lithium acetate transformation protocol (Yeast Protocols Handbook PT3024-1; Clontech) and cultured at 30 °C on SD/-Leu-Trp for 2 days. The transformed yeast colonies were suspended in sterilized water, OD600 = 1.0 was adjusted, and then diluted on SD/-Leu-Trp, SD/-Leu-Trp-Ade-His and SD/-Leu-Trp-Ade-His with 20 mM 3AT media according to 10^–1^, 10^–2^ and 10^–3^ gradient, and were checked and photographed after culture at 30 °C for 2 days.

The β-galactosidase activity was determined using the methods described previously (Parveen et al. 2023). The transformed yeasts were cultured at 30 °C in YPDA liquid medium for 2 days. Yeast cells were gathered and suspended in 600 µl of Z-buffer (60 mM Na_2_HPO_4_, 40 mM NaH_2_PO_4_, 10 mM KCl, 1 mM MgSO_4_, 50 mM β‐mercaptoethanol, pH 7.0). The suspension was then placed in liquid nitrogen for 1 min, followed by 37 °C for 3 min, and this process was repeated four times. After equilibration at 30 °C for 5 min, 120 µl of 4 mg/ml o‐nitrophenyl‐β‐d‐galactoside was added to the mixture and thoroughly vortexed before incubation at 30 °C. The reaction was then stopped by adding 400 µl of 1 M Na_2_CO_3_. The relative β‐galactosidase activity unit was calculated as follows: (1000 × D_420nm_)/(D_600nm_ × reaction time (min) × 0.1 × dilution factor).

### Primer Sequences

All the oligo sequences used in this study were listed in Table S1.

## Supplementary Information

Supplementary material 1: Subcellular localization of ROC1-8 proteins. GFP signals of the ROC1-8-GFP and GFP control after infiltration into *N.*  *benthamiana* leaves. The nucleus was visualized by DAPI staining (bar = 50 µm).

Supplementary material 2: The relative expression of ROC1-8 genes in ZH11 flag leaves. Bars represent the SD of measurements (n = 3).

Supplementary material 3: Alignment of ROC1 and ROC8 protein sequences to their respective Crisper-CAS9 edited protein sequences. **A** Alignment of ROC1 protein sequence to Crisper-CAS9 edited ROC1 proteins in ROC1KO-1, ROC1KO-13, ROC1/8DKO-12 and ROC1/8DKO-16 plants. **B** Alignment of ROC8 protein sequence to Crisper-CAS9 edited ROC8 proteins in ROC1KO-1, ROC1KO-13, ROC1/8DKO-12 and ROC1/8DKO-16 plants. The translation of ROC1 and ROC8 proteins is prematurely terminated following Crisper-CAS9 editing.

Supplementary material 4: Status of some plants before drought treatment. **A** Status of ZH11, ROC1KO-1 and ROC1KO-13 plants before direct water cut-off treatment. **B** Status of ZH11, ROC1KO-1 and ROC1KO-13 plants before 20% PEG6000 treatment. **C** Status of ROC1KO-1, ROC1/8DKO-12 and ROC1/8DKO-16 plants before direct water cut-off treatment. **D** Status of ROC1KO-1, ROC1/8DKO-12 and ROC1/8DKO-16 plants before 20% PEG6000 treatment.

Supplementary material 5: The drought tolerance detection of the ROC1OE plants and WT ZH11 plants. **A** Status of the ZH11, ROC1OE-3 and ROC1OE-5 plants before direct water cut-off treatment. **B** The status of the ZH11, ROC1OE-3 and ROC1OE-5 plants after water cut-off treatment for 7 days. **C** The status of the ZH11, ROC1OE-3 and ROC1OE-5 plants after re-watering for 2 days. **D** The survival rates of the plants in C. **E** Status of ZH11, ROC1OE-3 and ROC1OE-5 plants before 20% PEG6000 treatment. **F** The status of the ZH11, ROC1OE-3 and ROC1OE-5 plants after 20% PEG6000 treatment for 11 days. **G** The status of the ZH11, ROC1OE-3 and ROC1OE-5 plants after re-watering for 2 days. **H** The survival rates of the plants in G. Bars represent the SD of measurements (n = 3).

Supplementary material 6: ROC1 interacts with ROC1, ROC5 and ROC8 proteins verified by Y2H assays. The transformed yeast grows on the SD-Leu-Trp and SD-Ade-His-Leu-Trp media.

Supplentary material 7: Number and length of stomata on the adaxial surface of the leaves of the ROC1KO and ROC1OE lines in comparison with those of the WT ZH11. **A** SEM of the adaxial surface of ZH11 leaf. **B** SEM of the adaxial surface of ROC1KO-1 leaf. **C** SEM of the adaxial surface of ROC1KO-13 leaf. **D** SEM of the adaxial surface of ROC1OE-3 leaf. **E** SEM of the adaxial surface of ROC1OE-5 leaf. The red asterisks represent the stomata and bars = 20 µm in **A**–**E**. **F** Average stomata numbers per square millimeter calculated from 5 plants for each line. **G** The stomata length of ZH11, ROC1KO-1, ROC1KO-13, ROC1OE-3 and ROC1OE-5 plants. Data are means ± SD in **F** and **G**.

Supplementary material 8: Sequences of primers used in this study.

## Data Availability

The datasets used and/or analyzed during the current study are available from the corresponding author on reasonable request.

## Abbreviations

HD-ZIP IV: Homeodomain leucine zipper IV
TFs: Transcription factors
DAPI: 4',6-Diamidino-2-phenylindole
GL2: GLABRA2
ROC1-9: Rice outermost cell-specific 1-9
qRT–PCR: Quantitative real–time RT–PCR
LRI: Leaf rolling index
BiFC: Bimolecular fluorescence complementation
Co-IP: Co-immunoprecipitation assay
LCI: Luciferase complementation imaging assay
Y2H: Yeast two-hybrid assay
