# Correction: HD-ZIP IV Gene *ROC1* Regulates Leaf Rolling and Drought Response Through Formation of Heterodimers with ROC5 and ROC8 in Rice

**DOI:** 10.1186/s12284-024-00738-4

**Published:** 2024-09-09

**Authors:** Zhihuan Tao, Xuexia Miao, Zhenying Shi

**Affiliations:** 1 Key Laboratory of Plant Design, CAS Center for Excellence in Molecular Plant Sciences, Institute of Plant Physiology and Ecology, Chinese Academy of Sciences, Shanghai, 200032 China; 2 University of Chinese Academy of Sciences, Beijing, 100049 China

**Correction: Rice (2024) 17:45**

10.1186/s12284-024-00717-9

In this article, the citation of (Li, et al., 2010) should be ,

Li L, Shi Z.Y, Li L, Shen GZ, Wang XQ, An LS, Zhang JL (2010) Overexpression of ACL1 (abaxially curled leaf 1) increased Bulliform cells and induced Abaxial curling of leaf blades in rice. Mol Plant, 3(5): 807–817. doi: 10.1093/mp/ssq022.

but published incorrectly as below,

Li H, Liang W, Jia R, Yin C, Zong J, Kong H, Zhang D (2010) The AGL6-like gene OsMADS6 regulates floral organ and meristem identities in rice. Cell research 20 (3):299–313. doi:10.1038/cr.2009.14.

The original article has been corrected.

