# Supplementary figures and images for: HD-ZIP IV Gene ROC1 Regulates Leaf Rolling and Drought Response Through Formation of Heterodimers with ROC5 and ROC8 in Rice
Source: Rice (N Y). 2024 Jul 27;17:45. doi: 10.1186/s12284-024-00717-9 (PMC11282044; doi:10.1186/s12284-024-00717-9)

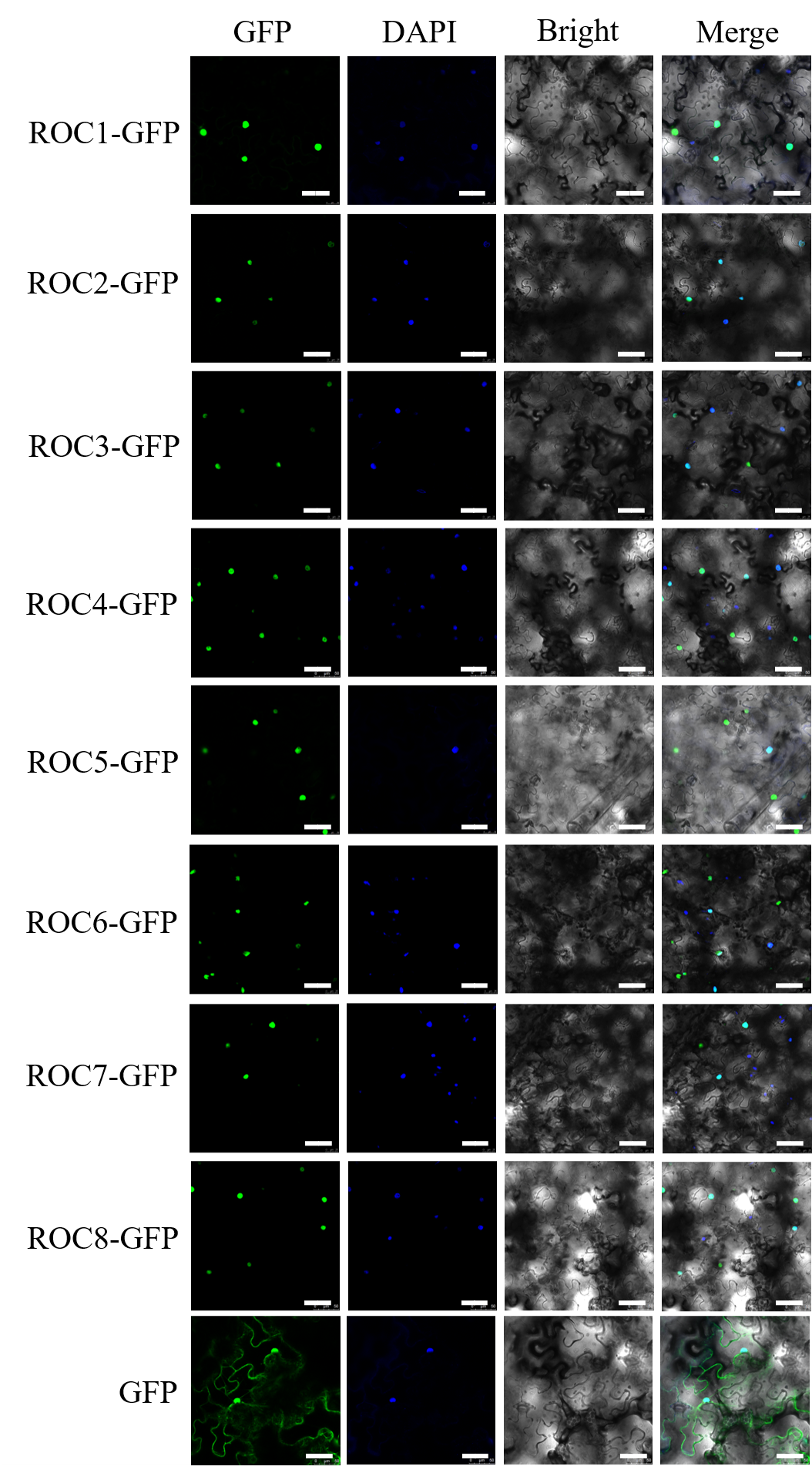

Supplement: Supplementary file 1 — Supplementary material 1: Subcellular localization of ROC1-8 proteins. GFP signals of the ROC1-8-GFP and GFP control after infiltration into N. benthamiana leaves. The nucleus was visualized by DAPI staining (bar = 50 µm). [file 12284_2024_717_MOESM1_ESM.tif]

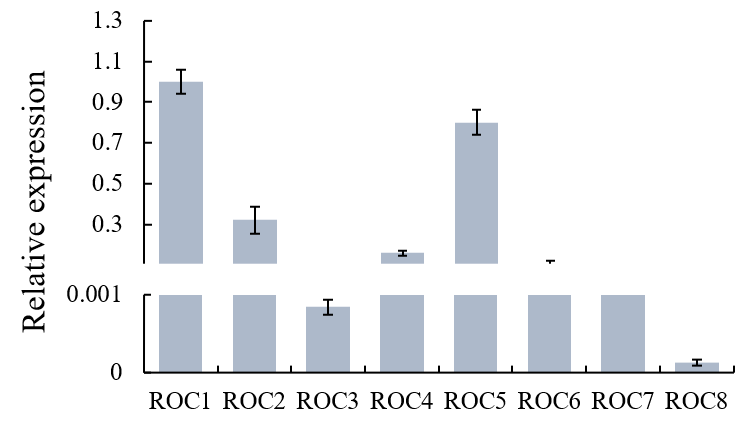

Supplement: Supplementary file 2 — Supplementary material 2: The relative expression of ROC1-8 genes in ZH11 flag leaves. Bars represent the SD of measurements (n = 3). [file 12284_2024_717_MOESM2_ESM.tif]

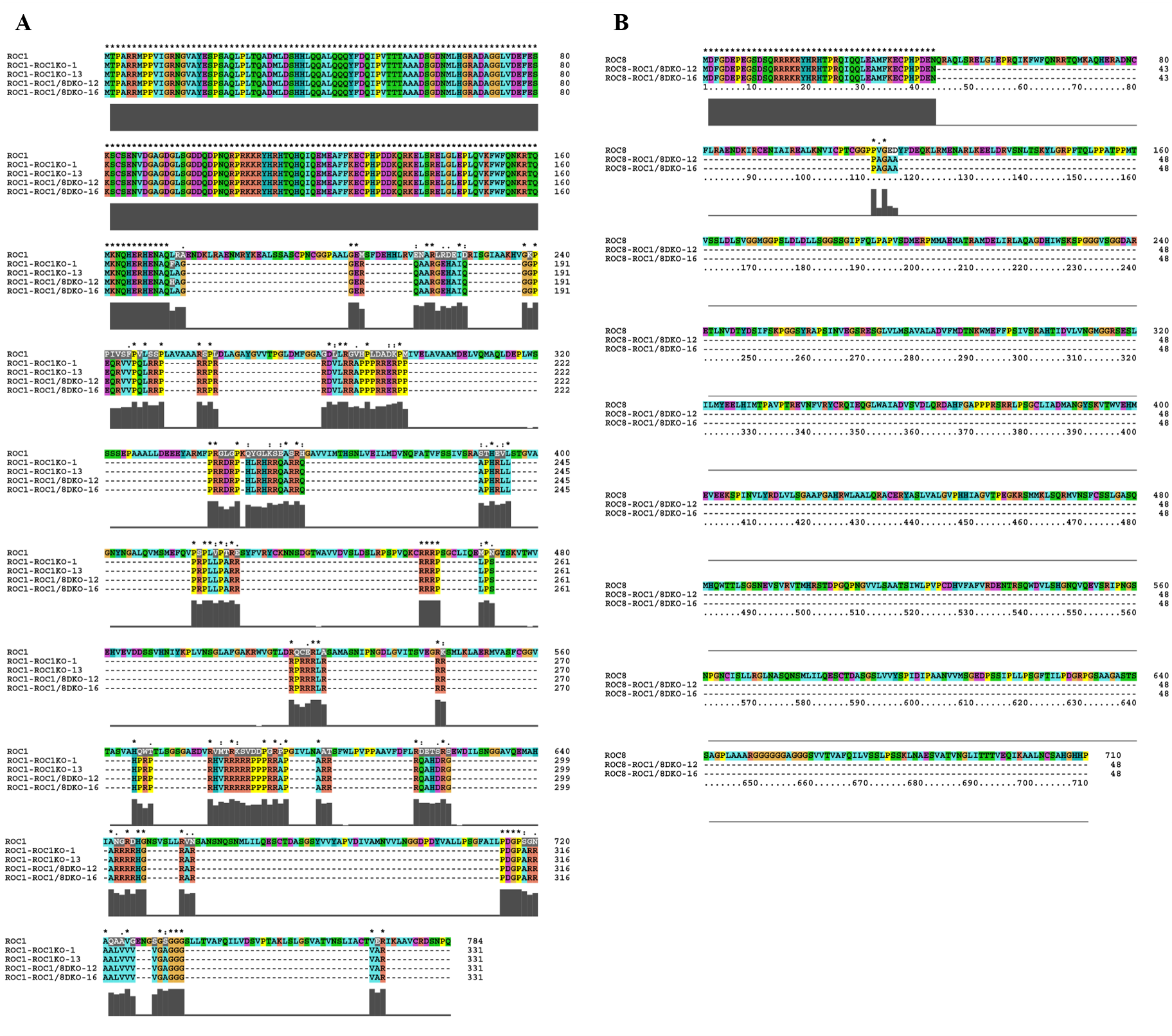

Supplement: Supplementary file 3 — Supplementary material 3: Alignment of ROC1 and ROC8 protein sequences to their respective Crisper-CAS9 edited protein sequences. A Alignment of ROC1 protein sequence to Crisper-CAS9 edited ROC1 proteins in ROC1KO-1, ROC1KO-13, ROC1/8DKO-12 and ROC1/8DKO-16 plants. B Alignment of ROC8 protein sequence to Crisper-CAS9 edited ROC8 proteins in ROC1KO-1, ROC1KO-13, ROC1/8DKO-12 and ROC1/8DKO-16 plants. The translation of ROC1 and ROC8 proteins is prematurely terminated following Crisper-CAS9 editing. [file 12284_2024_717_MOESM3_ESM.tif]

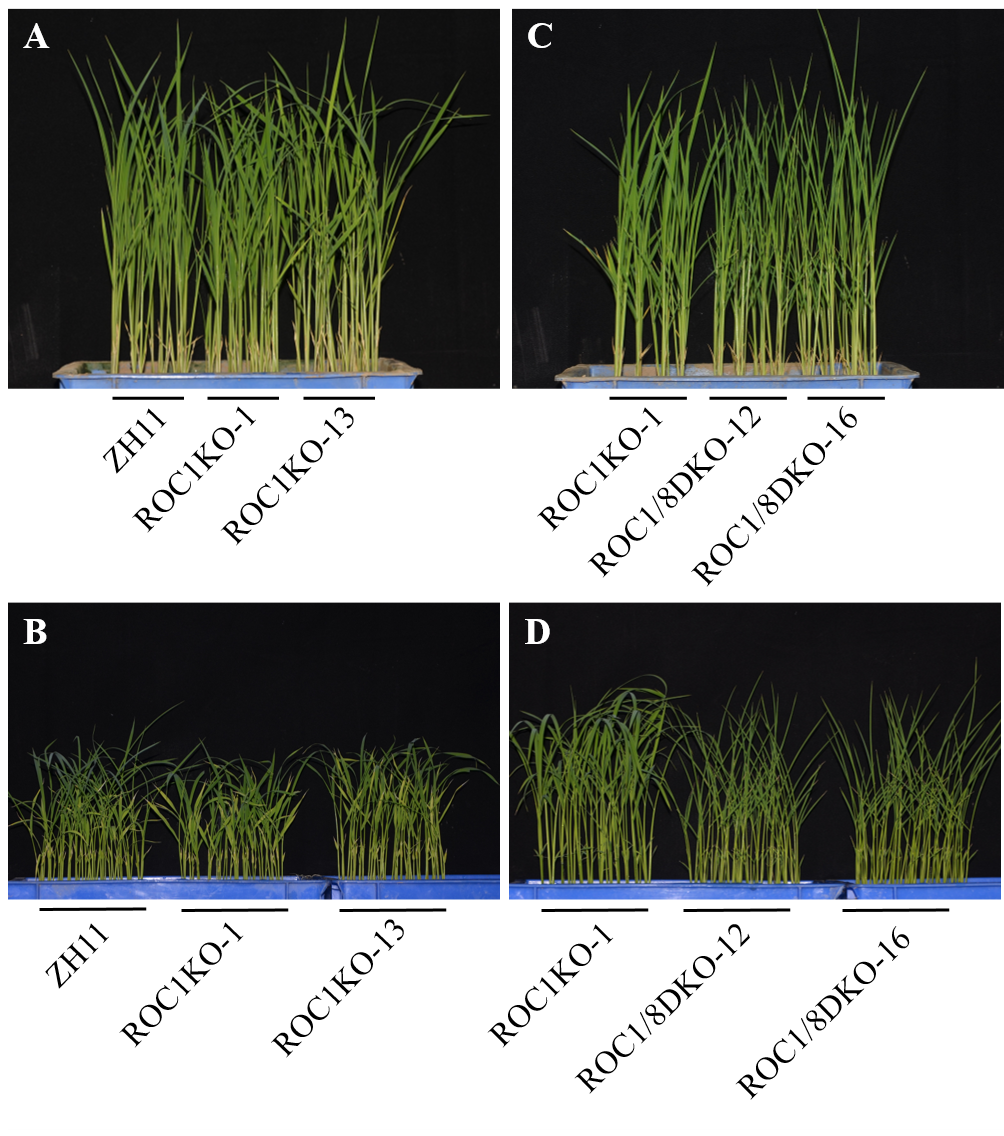

Supplement: Supplementary file 4 — Supplementary material 4: Status of some plants before drought treatment. A Status of ZH11, ROC1KO-1 and ROC1KO-13 plants before direct water cut-off treatment. B Status of ZH11, ROC1KO-1 and ROC1KO-13 plants before 20% PEG6000 treatment. C Status of ROC1KO-1, ROC1/8DKO-12 and ROC1/8DKO-16 plants before direct water cut-off treatment. D Status of ROC1KO-1, ROC1/8DKO-12 and ROC1/8DKO-16 plants before 20% PEG6000 treatment. [file 12284_2024_717_MOESM4_ESM.tif]

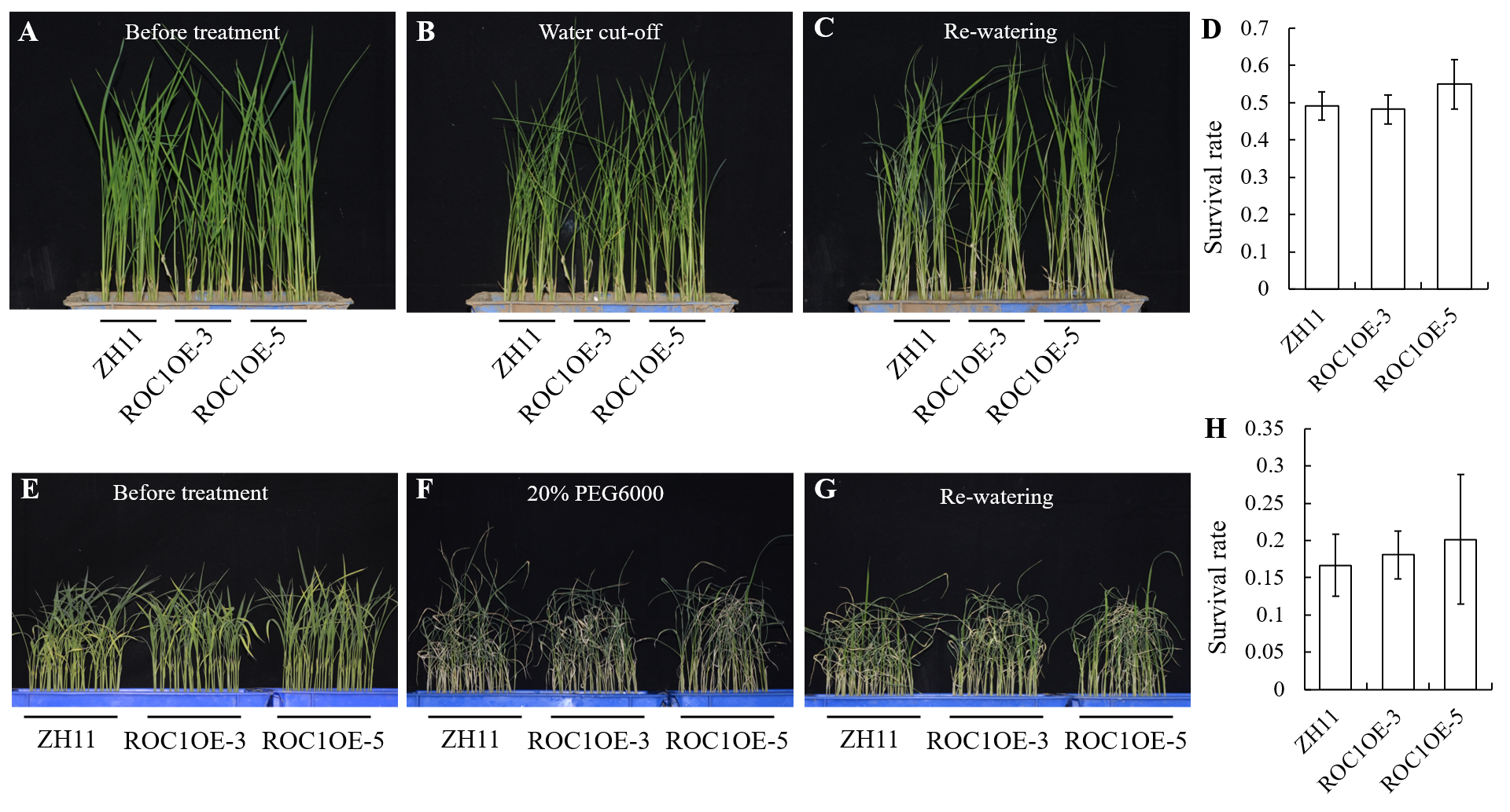

Supplement: Supplementary file 5 — Supplementary material 5: The drought tolerance detection of the ROC1OE plants and WT ZH11 plants. A Status of the ZH11, ROC1OE-3 and ROC1OE-5 plants before direct water cut-off treatment. B The status of the ZH11, ROC1OE-3 and ROC1OE-5 plants after water cut-off treatment for 7 days. C The status of the ZH11, ROC1OE-3 and ROC1OE-5 plants after re-watering for 2 days. D The survival rates of the plants in C. E Status of ZH11, ROC1OE-3 and ROC1OE-5 plants before 20% PEG6000 treatment. F The status of the ZH11, ROC1OE-3 and ROC1OE-5 plants after 20% PEG6000 treatment for 11 days. G The status of the ZH11, ROC1OE-3 and ROC1OE-5 plants after re-watering for 2 days. H The survival rates of the plants in G. Bars represent the SD of measurements (n = 3). [file 12284_2024_717_MOESM5_ESM.tif]

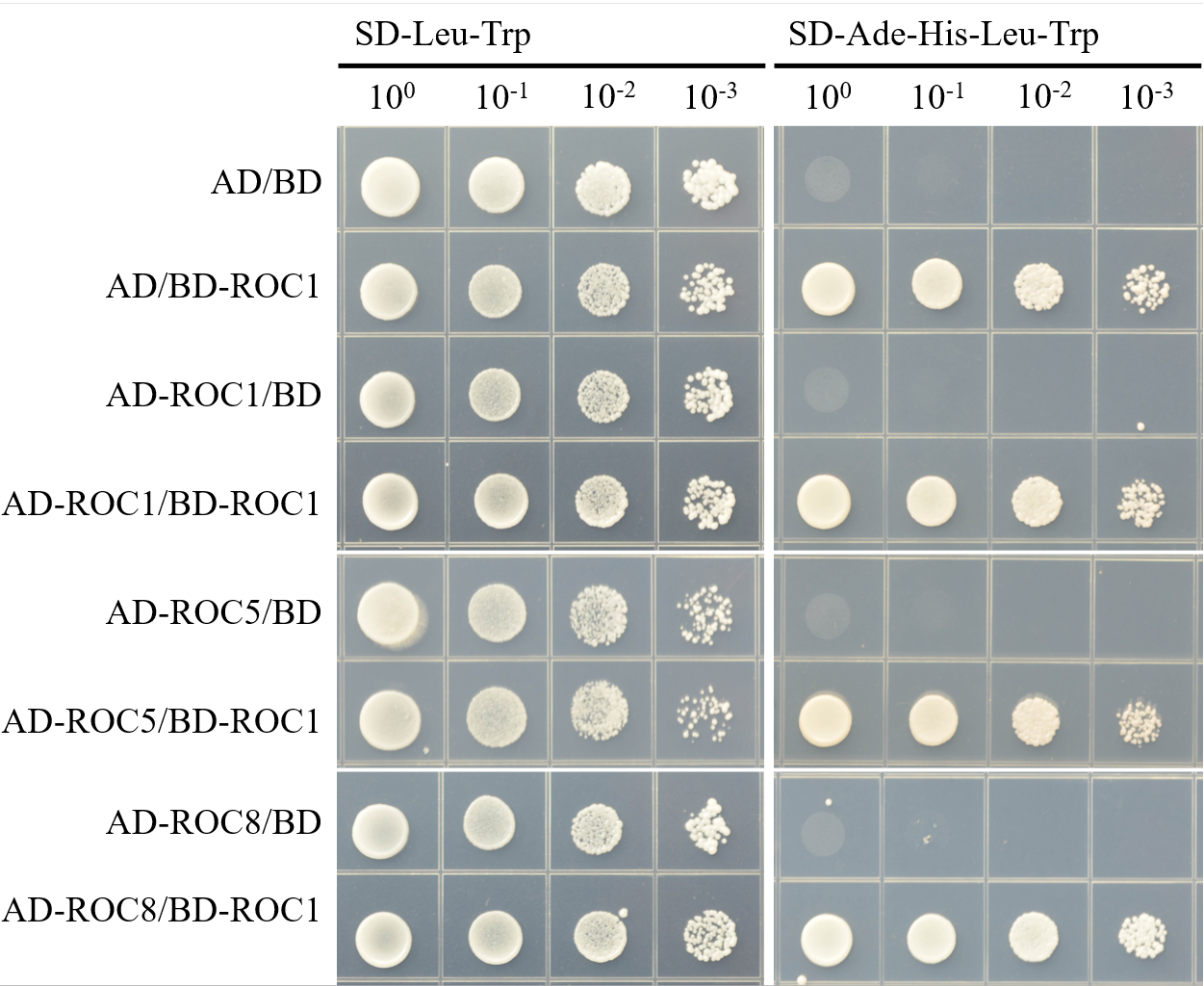

Supplement: Supplementary file 6 — Supplementary material 6: ROC1 interacts with ROC1, ROC5 and ROC8 proteins verified by Y2H assays. The transformed yeast grows on the SD-Leu-Trp and SD-Ade-His-Leu-Trp media. [file 12284_2024_717_MOESM6_ESM.tif]

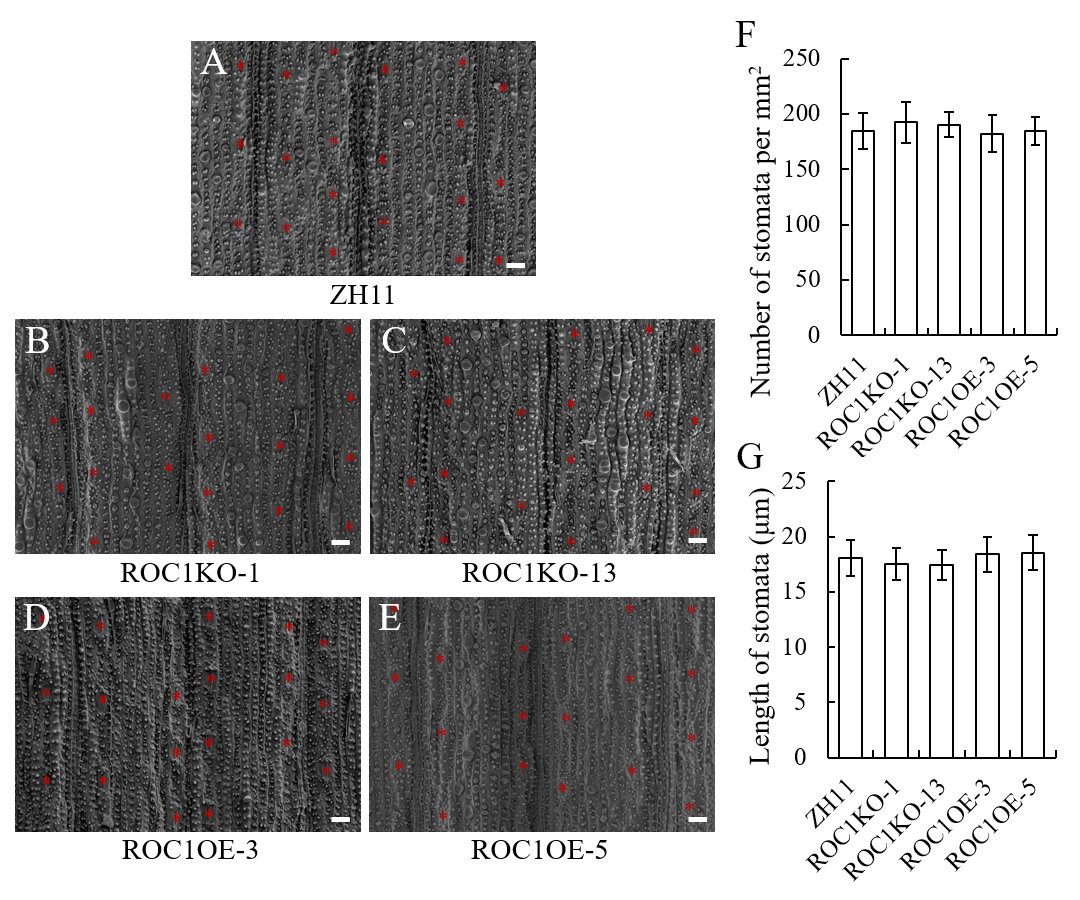

Supplement: Supplementary file 7 — Supplentary material 7: Number and length of stomata on the adaxial surface of the leaves of the ROC1KO and ROC1OE lines in comparison with those of the WT ZH11. A SEM of the adaxial surface of ZH11 leaf. B SEM of the adaxial surface of ROC1KO-1 leaf. C SEM of the adaxial surface of ROC1KO-13 leaf. D SEM of the adaxial surface of ROC1OE-3 leaf. E SEM of the adaxial surface of ROC1OE-5 leaf. The red asterisks represent the stomata and bars = 20 µm in A–E. F Average stomata numbers per square millimeter calculated from 5 plants for each line. G The stomata length of ZH11, ROC1KO-1, ROC1KO-13, ROC1OE-3 and ROC1OE-5 plants. Data are means ± SD in F and G. [file 12284_2024_717_MOESM7_ESM.tif]
